# Management of a Complex Case of Primary Enuresis in an Adult With Attention-Deficit Hyperactivity Disorder: A Case Report

**DOI:** 10.7759/cureus.79376

**Published:** 2025-02-20

**Authors:** Idriss A Mohamed, Faiza Ejas, Sameer A Khan, Amina Mujahid, Faisal A Nawaz

**Affiliations:** 1 Psychiatry, Al Amal Psychiatric Hospital, Emirates Health Services, Dubai, ARE; 2 Anesthesia and Critical Care, College of Medicine, Mohammed Bin Rashid University of Medicine and Health Sciences, Dubai Heath, Dubai, ARE; 3 Internal Medicine, College of Medicine, Mohammed Bin Rashid University of Medicine and Health Sciences, Dubai Heath, Dubai, ARE; 4 Cardiology, College of Medicine, Mohammed Bin Rashid University of Medicine and Health Sciences, Dubai Heath, Dubai, ARE

**Keywords:** attention deficit hyperactivity disorder (adhd), bipolar affective disorder, borderline personality disorder (bpd), methylphenidate (mph), primary enuresis

## Abstract

Enuresis is the inability to maintain voluntary control over urination, which is a relatively uncommon condition in adults. Therefore, there is limited research exploring the management of primary enuresis in adult patients with comorbid attention-deficit hyperactivity disorder (ADHD), particularly in the Middle Eastern region. We report the case of a 28-year-old female patient who has been following up and treated for comorbid bipolar affective disorder (BAD) and borderline personality disorder (BPD) for 12 years, complicated with primary enuresis. She was treated with 10 mg oral escitalopram once daily and a monthly intramuscular injection of 400 mg aripiprazole extended-release, with no reported improvement in her symptoms. Upon recent comprehensive reassessment, the patient was found to have a missed diagnosis of ADHD. Along with her current medications, she was started on 18 mg methylphenidate once daily and lamotrigine, started by 25 mg once daily, titrated to 50 mg. This adjustment led to significant improvement in her symptoms and the resolution of her enuresis. This case report demonstrates the resolution of ADHD symptoms along with the primary enuresis in an adult female patient who was treated with methylphenidate.

## Introduction

Attention-deficit hyperactivity disorder (ADHD) is a prevalent neurodevelopmental disorder, impacting an estimated 139.8 million individuals globally [1]. It is characterized by a triad of inattention, hyperactivity, and impulsivity [2]. Enuresis, commonly referred to as bedwetting, involves the involuntary act of urination. Its prevalence usually diminishes with age. At the age of five, 23% of individuals experience enuresis, while in adulthood, the prevalence decreases to 0.5-2% [3].

As per the Diagnostic and Statistical Manual of Mental Disorders, Fifth Edition (DSM-5), enuresis is divided into primary and secondary enuresis [2]. Primary enuresis refers to cases where consistent nighttime dryness has never been achieved for a continuous period of six months [4]. In contrast, secondary enuresis occurs when bedwetting resumes after a dry period of at least six months. Enuresis is further divided into nocturnal and diurnal types. Nocturnal enuresis involves involuntary wetting during sleep or at night beyond the age when bladder control is generally expected, typically around five years. This differs from diurnal enuresis, which is characterized by involuntary urine loss while awake [5].

The correlation between enuresis and ADHD is widely recognized. About 40% of children dealing with enuresis also have a diagnosis of ADHD [6]. The thalamus, an essential area of the brain involved in arousal, exhibits reduced connectivity with various brain regions in children with enuresis, potentially contributing to arousal disorders. This dysfunction may also play a role in ADHD, where both arousal and attention regulation are disrupted [7].

This emphasizes the importance of routinely evaluating enuresis during ADHD assessments and vice versa, necessitating collaboration between psychiatrists and healthcare professionals. Unfortunately, there is a lack of research exploring the link between these conditions in adults and their prevalence.

Managing enuresis in individuals with ADHD demands a flexible approach, which can span from behavioral therapies to pharmaceutical guidance. Currently, the primary treatments for primary enuresis involve alarm systems (behavioral) and desmopressin tablets (pharmacological). The effectivity of these two mainstays is explored in a randomized cross-over study by Kwak et al. in which 77.8% of the desmopressin group and 82% of the enuresis alarm group achieved symptom improvement [8]. However, note that treating enuresis in conjunction with ADHD is more challenging due to issues with non-compliance. The purpose of examining this case is to highlight that the timely diagnosis of ADHD and its treatment using methylphenidate not only resolves ADHD symptoms but can potentially play an important role in managing concurrent primary enuresis.

## Case presentation

This case involves a 28-year-old female who has received treatment for bipolar affective disorder (BAD) and comorbid borderline personality disorder (BPD) for the past 12 years. She had associated symptoms of dysthymia, affective dysregulation, impulsivity, poor attention and focus, episodes of anger and verbal aggression. In addition to this, she reported involuntary bed-wetting incidents since birth that caused her to isolate herself due to shame and embarrassment. 

She was initially treated with escitalopram 10 mg once daily for four years, after which intramuscular aripiprazole extended-release 400 mg was added to her regimen for the following eight years. She also received Cognitive Behavioral Therapy (CBT) during this time, however, this treatment plan did not help alleviate her symptoms, and instead made her drowsy, thereby affecting her academic and social life. 

Regarding her primary enuresis, her mother reports that she has experienced daily episodes of involuntary bedwetting both during the day and night despite receiving both pharmacological (imipramine and desmopressin) and behavioral (alarm systems) treatments which unfortunately did not relieve her symptoms. No established diagnosis or family history of enuresis was present. 

The patient denies any history of childhood trauma or comorbid medical conditions. No previous history of admission to a psychiatric ward was recorded despite her having a coexisting diagnosis of BAD.

The patient reported a history of psychiatric conditions in the family, though she was unable to specify the exact conditions. Current ongoing stressors included a dysfunctional family dynamic with her parents due to her impulsivity and affective dysregulation. 

On the most recent visit, in 2023, her symptoms on follow-up did not meet the DSM-5 criteria for generalized anxiety disorder, or any bipolar mood symptoms in terms of mania or hypomania. She was then comprehensively screened for the symptoms of ADHD and the patient demonstrated the following symptoms of difficulty sustaining attention and increased number of “careless” errors at work as well as misplacing items and forgetting dates and tasks easily. The patient’s mother also described her to be an “impulsive and restless” person throughout her childhood. Considering the history of current symptoms, and collateral history from the patient’s family, a provisional diagnosis of ADHD was established, and the patient was immediately started on methylphenidate 18 mg once daily. 

Since the initiation of the current treatment, the patient reported no further episodes of bedwetting during her first and second-month follow-up appointments. Her mother also observed a significant improvement in her attention span and an increased ability to focus on tasks for extended periods without distraction. Additionally, there was noticeable improvement in the patient’s mood symptoms and a significant reduction in impulsive behavior during this time 

Investigations

The patient was clinically assessed to rule out other organic pathologies. Her complete physical examination and routine laboratory investigations were normal. The mental state examination was unremarkable other than restlessness on earlier presentation. Risk assessment revealed no apparent risk to self or others; the patient reported no suicidal or homicidal thoughts. 

Differential diagnosis

Differential diagnosis was ADHD, BAD, and borderline personality disorder, along with primary enuresis. 

Treatment and follow-up

**Figure 1:**
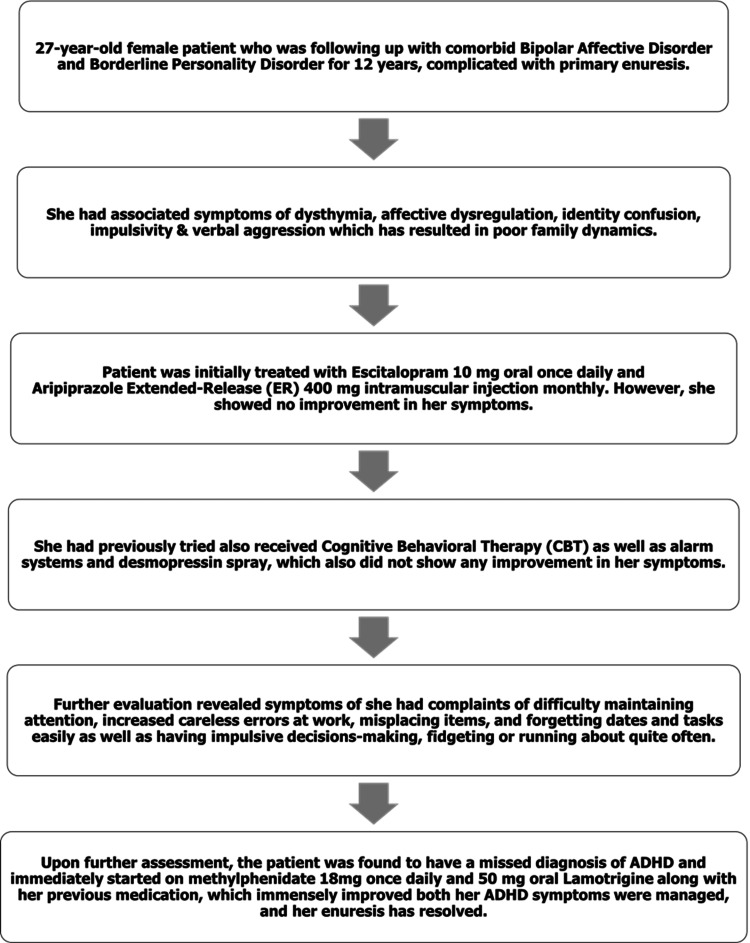
Patient management timeline ADHD: Attention-Deficit Hyperactivity Disorder

With the initial diagnosis of BAD co-morbid with BPD, the patient was started on 400 mg aripiprazole intramuscularly every month for the past eight years and 10 mg escitalopram oral daily for the past 12 years. Following the ADHD diagnosis, a new treatment plan was started by keeping her on the same dose of 10 mg oral escitalopram daily, started on 25 mg oral lamotrigine daily, and 18 mg methylphenidate daily at night. Her inattention symptoms showed remarkable improvement, and the dosage of the lamotrigine was then increased to 50 mg oral daily to reach the optimal dose for better management of her dysthymic mood. A few months later, methylphenidate was increased to 36 mg daily and lamotrigine gradually increased to 100 mg daily in the morning, however the patient started to experience headaches and therefore the mediations were lowered back to 18 mg methylphenidate and 50 mg lamotrigine. Aripiprazole 5 mg tablets were initially introduced to facilitate cross-tapering and gradually discontinue the intramuscular aripiprazole 400 mg. The 5 mg tablets were subsequently discontinued as part of the treatment plan. The patient's timeline of treatment is summarized in Figure 1.

## Discussion

This is the first case in the Middle East region, and the second case worldwide to be documented of an adult patient with primary enuresis and co-morbid ADHD to be managed with methylphenidate. Currently, there is a lack of literature regarding primary enuresis in adults. 

This case also aims to shed light on the underdiagnosis of ADHD in adults and how it can be further complicated by the complexity of additional symptoms from comorbid conditions such as BAD as both disorder symptoms overlap. Undiagnosed ADHD in adults can significantly impact their daily lives and can be misdiagnosed with bipolar disorder which eventually will lead to adverse effects in their physical and mental health if not detected and appropriately treated at the earliest. 

Additionally, this case also highlights the critical importance of psychiatrists honing their diagnostic skills to differentiate between ADHD and bipolar disorder, as these distinct conditions require different treatment approaches. 

Exploring the missed diagnosis of ADHD

This case highlighted the prevalence of undiagnosed ADHD in adults in the background of underlying social dysfunction. Despite its prevalence, research has shown that adults with ADHD often get misdiagnosed or are not diagnosed at all.

Faraone et al. (2004) conducted a medical review on adults with ADHD and reported that only 25% of adult ADHD cases were diagnosed in childhood or adolescence [9].

Studies have shown that a major cause of difficulty in the diagnosis of ADHD is due to sex differences. A 2011 study found that ADHD prevalence is higher in men than in women 2.28:1, and a study conducted in Sweden by Mowlem et al. in 2018 concluded that female patients may be more easily missed in the ADHD diagnostic process and less likely to be prescribed medication unless they have prominent externalizing problems, despite clinically diagnosed males and females showing similar symptom severity [10,11].

Another major factor is the age of the patient at diagnosis. Research has found significantly higher rates of diagnosis among children who are born just before the school entry cut-off date, indicating that relative maturity and developmental age are not consistently considered [12].

ADHD symptoms, characterized by poor concentration, physical restlessness, impulsivity, and mood fluctuations, have a tendency to overlap with manifestations seen in depression, anxiety, BAD, and borderline personality disorder [13,2]. This overlap poses the risk of misdiagnosis or diagnosis being missed entirely, where an incorrect assessment might be made. The complexity of ADHD is evident in its high comorbidity rate; approximately 75% of adults with ADHD also receive a concurrent psychiatric diagnosis, encompassing mood disorders, anxiety, personality disorders, and substance misuse [14]. Frequently, it is the associated conditions that garner attention, inadvertently leading to the oversight of ADHD symptoms

Overlap of symptoms between ADHD and BAD

Although ADHD is historically referred to as a neurodevelopmental syndrome specifically seen in children, recent studies have expanded to include the adult population in the picture. According to a meta-analysis that reviewed articles between 2005 to 2019, about 2.58% of the population had persistent adult ADHD [1]. Interestingly, adult ADHD and BAD have been found to be a common comorbidity in many cross-sectional studies which demonstrates that 5% to 47% of adult ADHD patients were found to have comorbid BAD [15].

It may be difficult to differentiate the symptoms of ADHD and BAD as they both can present with restlessness, talkativeness, distractibility, and impulsivity; however, ADHD lacks aspects of a manic episode such as grandiosity, elated mood, and decreased need for sleep as well as the episodic course of the disorder [16].

Possible mechanisms regarding the comorbidity of ADHD with BAD have been discussed stating that it could be a “true comorbidity” due to shared risk factors or overlap between risk factors which could tip the scale into either disorder, whether the comorbidity creates a separate meaningful syndrome on its own or even if one disorder may increase the risk for the other. On the other hand, it could be a result of “false comorbidity” due to an overlap in the diagnostic criteria, developmental sequencing or just referral bias [17].

One issue to consider in such cases is if patients require a “primary diagnosis” when both conditions are present. Youngstrom et al. suggest using either the diagnosis that comes developmentally first, the more severe diagnosis or the diagnosis that brings the patient into the clinic which can later be useful in management for the patient [18].

Concerning the treatment of comorbid bipolar and ADHD, the first goal should be mood stabilization. Some patients may see a resolution of their ADHD symptoms altogether while others still experience some residual effects such as memory loss or inattention which can prompt the physician to start stimulants such as methylphenidate or amphetamine. They are usually well tolerated but there is a cautionary chance of inducing a manic episode in certain patients [19].

Overlap of symptoms between ADHD and BPD

BPD is categorized as a personality disorder in the DSM-5, which describes it as a “pattern of instability in interpersonal relationships, self-image, and affects, along with marked impulsivity,” whereas ADHD is classified as a neurodevelopmental disorder, defined as “a persistent pattern of inattention and/or hyperactivity-impulsivity that disrupts functioning or development” [2].

Adult ADHD and BPD have been found to be comorbid as 9.5% to 21% of bipolar patients were found to have comorbid ADHD (Wingo and Ghaemi, 2007) [15]. Although both BPD and ADHD share a few features including impulsivity and emotion dysregulation, they are often seen in different contexts. For example, impulsivity in BPD is considered in the setting of self-harm and ADHD is in the setting of impatience. Furthermore, difficulties in impulse control have been linked to increased risk of enuresis, as impulsive behaviors may override the ability to recognize and respond to bodily signals [20]. This connection between emotional regulation and bodily control is further supported by neuroimaging studies done in women which have indicated that individuals with BPD show distinct neural responses related to emotional regulation, with reduced prefrontal cortex activity during emotional tasks. This neural dysregulation may hinder the ability to control bodily functions, contributing to enuresis [21].

A study by Kuja-Halkola et al. investigated the co-occurrence and familial co-aggregation of clinically ascertained ADHD and BPD diagnoses in the Swedish population which revealed that individuals diagnosed with ADHD were about 19 times more likely to also have a BPD diagnosis compared to those without ADHD. Additionally, having a sibling with ADHD increased the likelihood of being diagnosed with BPD, with the risk varying depending on the closeness of the genetic relationship, indicating potential shared genetic factors [22].

In terms of treatment for these conditions, Asherman et al. conducted a literature review that exposed the lack of established information and guidelines regarding the treatment of adults with ADHD and comorbid BPD. The study recommends dialectical behavioral therapy (DBT) for BPD and brings to light that it may also help manage ADHD symptoms. It also suggests that ADHD should always be considered when treating comorbid personality disorders to “reduce distress, improve daily functioning, and help patients engage better with psychological treatments for BPD” [23]. This is also demonstrated by Prada et al. [24] who found that the therapy outcome of BPD-ADHD patients who received methylphenidate showed a significantly improved response to DBT treatment when compared with those without it.

Methylphenidate efficacy for primary enuresis

Regarding the use of methylphenidate with enuresis and ADHD, the existing literature presents contrasting perspectives. On one side, some sources suggest that methylphenidate effectively addresses both enuresis and ADHD symptoms, while on the other side, there are reports indicating that individuals undergoing ADHD treatment with methylphenidate may experience nocturnal enuresis.

A study done by Ferrara et al. explores the efficacy of methylphenidate in addressing primary enuresis. Among the 103 ADHD patients, nine patients presented with comorbid nocturnal enuresis, these nine were treated with methylphenidate, and six out of eight showed notable improvements within six months [25]. Methylphenidate acts as an agonist on dopamine and norepinephrine receptors. Its ability to activate norepinephrine receptors accounts for the reduced bladder contractility and promotion of detrusor relaxation, contributing to decreased urinary incontinence [26]. Additionally, multiple studies have demonstrated that methylphenidate increases the maximum urethral closure pressure [27].

Conversely, literature with a contradictory viewpoint is evident in a case report by Ghanizadeh. The report talks about a case of an 11-year-old boy treated for ADHD with methylphenidate, resulting in the onset of nocturnal enuresis. The medication was discontinued, and upon reinitiating methylphenidate after three months, the same enuresis recurrence was observed [28]. Further research is warranted to explore the pharmacodynamic effects of methylphenidate on pediatric and adult age groups that are not responding to traditional first-line treatments.

Medication adjustments and their role in resolving enuresis

The role of medication adjustments in the resolution of enuresis in this case warrants careful consideration. The patient had been on escitalopram (10 mg once daily) for four years, after which intramuscular aripiprazole (400 mg extended release) was introduced and maintained for the next eight years. While there is substantial evidence linking serotonergic and atypical antidepressants to urinary incontinence, a direct association between escitalopram and enuresis is not well established in the literature [29]. In our case, the dose of escitalopram remained unchanged and lamotrigine was maintained at 50 mg, whereas aripiprazole was gradually tapered down and discontinued. Therefore, modification to these medications may have contributed to the resolution of enuresis, rather than the addition of methylphenidate alone. This highlights the complexity of factors influencing urinary continence in patients with ADHD, particularly in the context of polypharmacy.

Although there is no clear evidence on the role of lamotrigine in enuresis, drugs that depress dopamine activity have been associated with urinary incontinence, suggesting that lamotrigine's pharmacological profile could similarly disrupt normal voiding patterns [30].

Interestingly, aripiprazole has been shown to have beneficial effects in reducing enuresis. In a double-blind study, the frequency of enuresis was lower in the aripiprazole group compared to placebo (6.4% vs. 8%) after treatment initiation [31]. Additionally, case reports describe complete resolution of enuresis in children with psychiatric disorders following aripiprazole treatment [32]. These findings suggest that aripiprazole may have a protective effect against enuresis, making its discontinuation a potential factor in this case.

## Conclusions

The management of psychiatric and medical comorbidities can often have an overlap in the approach and treatment of complex presentations. The importance of timely diagnosis of ADHD can be life-changing for patients in improving their quality of life. Moreover, this case report demonstrates the resolution of ADHD symptoms along with the primary enuresis in an adult treated with methylphenidate. However, further studies are warranted to explore the role of these medications as well as other underlying factors in the prevention and management of enuresis in the psychiatric population. 

## References

[1] Song P, Zha M, Yang Q, Zhang Y, Li X, Rudan I (2021). The prevalence of adult attention-deficit hyperactivity disorder: a global systematic review and meta-analysis. J Glob Health.

[2] (2022). Diagnostic and Statistical Manual of Mental Disorders: DSM-5-TR. Diagnostic and Statistical Manual of Mental Disorders: DSM-5-TR.

[3] (2024). Enuresis: Practice Essentials, Background, Pathophysiology. https://emedicine.medscape.com/article/1014762-overview.

[4] Daley SF, Rincon MG, Leslie SW (2024). Enuresis. StatPearls.

[5] Chin A, Lerman S (2008). Bedwetting. Encyclopedia of Infant and Early Childhood Development.

[6] Abd-Elmoneim N, Elsheshtawy E, Elsayed M (2020). Comorbidity between enuresis and attention deficit hyperactivity disorder: a case-control study. Middle East Curr Psychiatry.

[7] Zhang A, Zhang L, Wang M (2021). Functional connectivity of thalamus in children with primary nocturnal enuresis: results from a resting-state fMRI study. Brain Imaging Behav.

[8] Kwak KW, Lee YS, Park KH, Baek M (2010). Efficacy of desmopressin and enuresis alarm as first and second line treatment for primary monosymptomatic nocturnal enuresis: prospective randomized crossover study. J Urol.

[9] Faraone SV, Spencer TJ, Montano CB, Biederman J (2004). Attention-deficit/hyperactivity disorder in adults: a survey of current practice in psychiatry and primary care. Arch Intern Med.

[10] Ramtekkar UP, Reiersen AM, Todorov AA, Todd RD (2010). Sex and age differences in attention-deficit/hyperactivity disorder symptoms and diagnoses: implications for DSM-V and ICD-11. J Am Acad Child Adolesc Psychiatry.

[11] Mowlem FD, Rosenqvist MA, Martin J, Lichtenstein P, Asherson P, Larsson H (2019). Sex differences in predicting ADHD clinical diagnosis and pharmacological treatment. Eur Child Adolesc Psychiatry.

[12] Ford-Jones PC (2015). Misdiagnosis of attention deficit hyperactivity disorder: 'normal behaviour' and relative maturity. Paediatr Child Health.

[13] Barkley RA, Fischer M, Smallish L, Fletcher K (2002). The persistence of attention-deficit/hyperactivity disorder into young adulthood as a function of reporting source and definition of disorder. J Abnorm Psychol.

[14] Kooij SJ, Bejerot S, Blackwell A (2010). European consensus statement on diagnosis and treatment of adult ADHD: The European Network Adult ADHD. BMC Psychiatry.

[15] Wingo AP, Ghaemi SN (2007). A systematic review of rates and diagnostic validity of comorbid adult attention-deficit/hyperactivity disorder and bipolar disorder. J Clin Psychiatry.

[16] Katzman MA, Bilkey TS, Chokka PR, Fallu A, Klassen LJ (2017). Adult ADHD and comorbid disorders: clinical implications of a dimensional approach. BMC Psychiatry.

[17] Caron C, Rutter M (1991). Comorbidity in child psychopathology: concepts, issues and research strategies. J Child Psychol Psychiatry.

[18] Youngstrom EA, Arnold LE, Frazier TW (2010). Bipolar and ADHD comorbidity: both artifact and outgrowth of shared mechanisms. Clin Psychol (New York).

[19] Salvi V, Ribuoli E, Servasi M, Orsolini L, Volpe U (2021). ADHD and bipolar disorder in adulthood: clinical and treatment implications. Medicina (Kaunas).

[20] Schaich A, Assmann N, Köhne S (2021). The mediating effect of difficulties in emotion regulation on the association between childhood maltreatment and borderline personality disorder. Eur J Psychotraumatol.

[21] Silvers JA, Hubbard AD, Biggs E (2016). Affective lability and difficulties with regulation are differentially associated with amygdala and prefrontal response in women with borderline personality disorder. Psychiatry Res Neuroimaging.

[22] Kuja-Halkola R, Lind Juto K, Skoglund C (2021). Do borderline personality disorder and attention-deficit/hyperactivity disorder co-aggregate in families? A population-based study of 2 million Swedes. Mol Psychiatry.

[23] Asherson P, Young AH, Eich-Höchli D, Moran P, Porsdal V, Deberdt W (2014). Differential diagnosis, comorbidity, and treatment of attention-deficit/hyperactivity disorder in relation to bipolar disorder or borderline personality disorder in adults. Curr Med Res Opin.

[24] Prada P, Nicastro R, Zimmermann J, Hasler R, Aubry JM, Perroud N (2015). Addition of methylphenidate to intensive dialectical behaviour therapy for patients suffering from comorbid borderline personality disorder and ADHD: a naturalistic study. Atten Defic Hyperact Disord.

[25] Ferrara P, Sannicandro V, Ianniello F, Quattrocchi E, Sbordone A, Petitti T, Mariotti P (2019). Attention-deficit/hyperactivity disorder and enuresis: a study about effectiveness of treatment with methylphenidate or desmopressin in a pediatric population. Minerva Pediatr.

[26] Bahali K, Ipek H, Uneri OS (2013). Methylphenidate and atomoxetine for treatment of nocturnal enuresis in a child with attention-deficit hyperactivity disorder. Eur Child Adolesc Psychiatry.

[27] Chang JH, Lee KY, Kim TB, Yoon SJ, Lee T, Kim KH (2011). Clinical and urodynamic effect of methylphenidate for the treatment of giggle incontinence (enuresis risoria). Neurourol Urodyn.

[28] Ghanizadeh A (2008). Methylphenidate-associated enuresis in attention deficit hyperactivity disorder. J Pediatr Urol.

[29] Votolato NA, Stern S, Caputo RM (2000). Serotonergic antidepressants and urinary incontinence. Int Urogynecol J Pelvic Floor Dysfunct.

[30] Ambrosini PJ (1984). A pharmacological paradigm for urinary incontinence and enuresis. J Clin Psychopharmacol.

[31] Kılınç S, Hergüner A, Hergüner S (2017). Cessation of nocturnal enuresis with aripiprazole. J Child Adolesc Psychopharmacol.

[32] Nardello R, Guccione F, Gliubizzi C, Marino A, Capizzi M, Mangano S (2021). Resolution of enuresis with aripiprazole in children with psychiatric disorders: two case reports. J Med Case Rep.

